# First report on the leafhopper genus *Balera* Young (Hemiptera, Cicadellidae, Typhlocybinae, Alebrini) from Argentina, and description of a new species

**DOI:** 10.3897/zookeys.352.6283

**Published:** 2013-11-19

**Authors:** María Inés Catalano, Susana L. Paradell, Christopher H. Dietrich

**Affiliations:** 1 Centro de Bioinvestigaciones, Departamento de Ciencias Básicas y Aplicadas, Universidad Nacional del Noroeste de la Provincia de Buenos Aires, Monteagudo 2772, Pergamino, Buenos Aires, 2700, Argentina. CONICET; 2 División Entomología, Facultad de Ciencias Naturales y Museo, Universidad Nacional de La Plata. Paseo del Bosque s/n, La Plata, Buenos Aires, 1900, Argentina. CIC; 3 Illinois Natural History Survey, Institute of Natural Resource Sustainability, University of Illinois, 1816 S. Oak Street, Champaign, Illinois, 61820, USA

**Keywords:** Auchenorrhyncha, identification, morphology, distribution

## Abstract

The genus *Balera* Young is reported for first time to Argentina and a new species is described, *Balera floripara* **sp. n.** Detailed morphological descriptions and illustrations of the new species and a key to males of known species are provided. *Habralebra amoena* is also recorded for the first time from Argentina.

## Abstract (es)

El género *Balera* Young es registrado por primera vez para Argentina y una nueva especie es descripta, *Balera floripara* **sp. n.** Se provee de detalladas descripciones morfológicas e ilustraciones de la nueva especie, y una clave de las especies conocidas. Además *Habralebra amoena* se registra por primera vez para Argentina.

## Introduction

The tribe Alebrini McAtee, 1926 is represented by 34 genera (Balme 2007), six of which were recorded previously from Argentina: *Habralebra* Young, 1952, *Rhabdotalebra* Young, 1952, *Omegalebra* Young, 1957, *Protalebra* Baker, 1899, *Protalebrella* Young, 1952 and *Relaba* Young, 1957 (Young 1957, Dworakowska 1994, Catalano et al. 2010).

The genus *Balera* Young include twelve species recorded from Bolivia, Brazil, Colombia, Ecuador, Panama, Trinidad and Tobago and Venezuela (Young 1952, 1957; Ruppel 1959; Freytag 1992; Dworakowska 1994 and Coelho et al. 2013).

The genus *Habralebra* Young is represented by twelve species recorded from Argentina, Bolivia, Brazil, Ecuador, Nicaragua, Panama and Puerto Rico. *Habralebra willinki* Young, 1957, *Habralebra trimaculata* (Gillette, 1898) and *Habralebra gillettei* Young, 1957 were known previously from Argentina (Young 1957).

Here, we record *Balera* from Argentina for the first time based on a new species, *Balera floripara*, and record *Habralebra amoena* for the first time from Argentina.

## Materials and methods

The specimens were collected with Malaise and mercury vapor lights traps in Misiones and Jujuy provinces. For morphological study of the genital structures, clearing was accomplished by immersion of the entire abdomen in a solution of 10% KOH at room temperature for several hours followed by several rinses with water. For illustration, genital structures were embedded in glycerin. The color pattern here described is the post-mortem coloration. In living or recently collected individuals the coloration may be more vivid relative to that of old preserved specimens. Morphological terminology follows Young (1952) and Dietrich (2005) for habitus and genitalia characters. Digital photographs were taken using a QImaging Micropublisher 3.3 digital camera mounted on an Olympus SZX12 stereomicroscope. The type-series of the new species are deposited in the entomological collections of the Museo de Ciencias Naturales de La Plata, Argentina (MLP) and the Illinois Natural History Survey, USA (INHS).

## Taxonomy

### Alebrini McAtee

#### Balera

Young

http://species-id.net/wiki/Balera

Balera Young, 1952: 25. Type species: *Dikraneura pellucida* (Osborn), by original designation.

##### Diagnosis.

The genus *Balera* can be distinguished by the following combination of characters: forewing with appendix not extending around wing apex; hindwing with submarginal vein distinct and free from apical wing margin; male sternal abdominal apodemes slender and elongate, usually capitate apically; pygofer produced posteriorly, occasionally forming an apical process; subgenital plates with single or double row of weak macrosetae; style sigmoid in lateral aspect; connective V or Y-shaped or triangular; aedeagus shaft with one or two pairs of apical or anteapical processes.

##### Key to males of *Balera* (modified from Coelho et al. 2013 to include the new species)

**Table:** 

1	Aedeagus without long, paired distal processes, with short lobes or keels, or apex bifurcate	2
1'	Aedeagus with one or more pairs of slender distal processes longer than shaft width	5
2(1)	Aedeagus inflated, shaft keeled laterally, with 3 apical lobes (Young 1957, fig. 12D)	*Balera pellucida* (Osborn)
2'	Aedeagus not inflated, shaft with or without keels, without apical lobes	3
3 (2)	Aedeagus bifurcated distally	4
3'	Aedeagus not bifurcated, with pair of lateral keels on apical half (Young 1957, figs 13C, D)	*Balera pusilla* Young
4 (3)	Pygofer without apical process; aedeagus with long bifurcated apex (Ruppel 1959, figs 2E, F, G)	*Balera bracata* Ruppel
4'	Pygofer with short apical process; aedeagus with short bifurcated apex (Figs 2B, F, G)	*Balera floripara* sp. n.
5 (1')	Aedeagus with one pair of apical processes	6
5'	Aedeagus with two or more pairs of apical processes	11
6 (5)	Style with acute apex	7
6'	Style with truncate apex (Freytag 1992, fig. 7)	*Balera obtusa* Freytag
7 (6)	Apices of aedeagal processes convergent, in ventral view, near stem base (Freytag 1992, fig. 17)	*Balera napoensis* Freytag
7'	Apices of aedeagal processes not convergent	8
8 (7')	Apex of aedeagus narrow and pointed in ventral view	9
8'	Apex of aedeagus not pointed in ventral view	10
9 (8)	Style slightly angled at midlength; pygofer long, with posterior margin produced, narrowly rounded (Freytag 1992, fig. 23, 24)	*Balera plagata* Freytag
9'	Style strongly angled at midlength; pygofer broad, with posterior margin weakly produced (Freytag 1992, figs 11, 12)	*Balera ecuadora* Freytag
10 (8')	Posterior margin of pygofer broadly rounded (Young 1957, fig. 12L)	*Balera caraguatae* Young
10'	Posterior margin of pygofer with acuminate process (Dworakowska 1994, fig 219)	*Balera signata* Dworakowska
11 (5')	Aedeagus with two pairs of apical processes	12
11'	Aedeagus with three pairs of apical processes (Coelho et al. 2013, figs 14, 15)	*Balera fiuzai* Coelho, Nessimian, Da-Silva
12 (11)	Dorsoapical pair of aedeagal processes longer than ventral pair (Freytag 1992, figs 1, 2)	*Balera myersi* Freytag
12'	Dorsoapical pair of aedeagal processes approximately same length as ventral pair (Young 1957, figs 13F, H)	*Balera emarginata* (Osborn)

#### Balera floripara sp. n.

http://zoobank.org/E1AEF78C-AD35-462C-9CE3-20FA2A0DE2A3

http://species-id.net/wiki/Balera_floripara

Figs 1A , 2A–G

##### Description.

**Figure 1.**
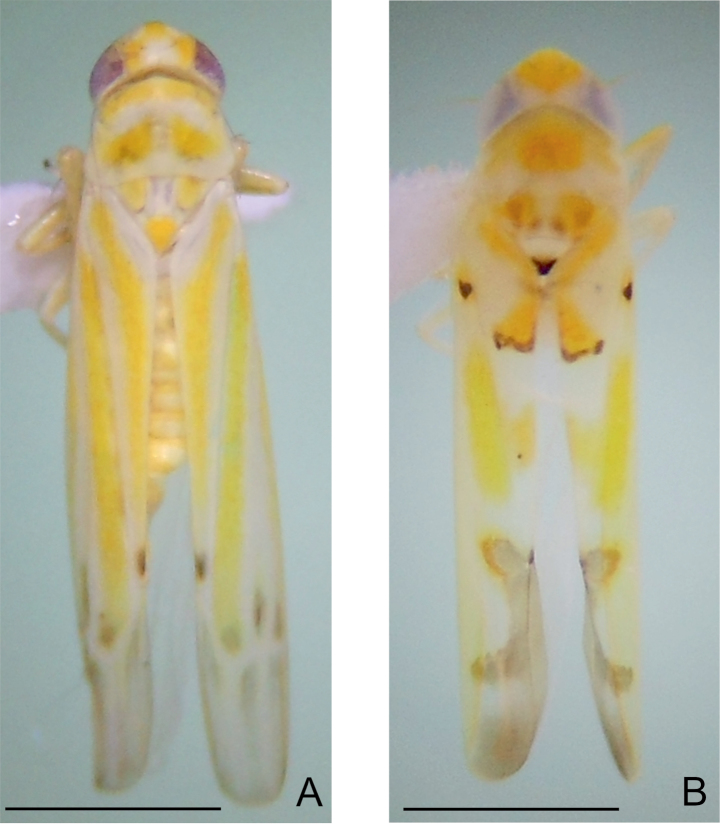
Dorsal habitus. **A** *Balera floripara* sp. n. **B** *Habralebra amoena*. Scale = 1 mm.

Length of male 3.7–3.8 mm. Ground color pale-yellow; crown, pronotum and scutellum with yellow markings; forewing with longitudinal yellow stripes on clavus and along CuA in corium, apical tip of clavus dark brown, with brown markings on bases of apical cells and apices of anteapical cells arranged in radial pattern (Fig. 1A).

**Figure 2.**
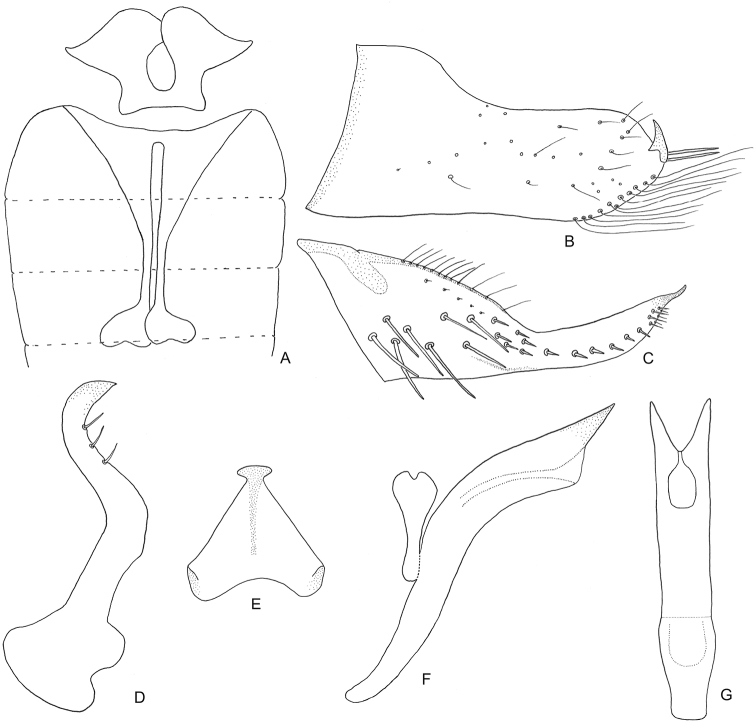
*Balera floripara* sp. n. Male (**A–G**) **A** sternal apodeme 1S and 2S **B** pygofer, lateral view **C** subgenital plate **D** style **E** connective **F** aedeagus in lateral view **G** aedeagus in posterior view.

Male: First sternal apodemes (1S) (Fig. 2A) enlarged with apices overlapping. Second sternal apodemes (2S) (Fig. 2A) slender and elongate, reaching sixth segment, apices capitate. Pygofer (Fig. 2B) with posterior margin produced, with short, acute apical process directed dorsad and row of very long, thin setae on postero-ventral margin. Subgenital plate (Fig. 2C), in lateral view, with basal half strongly tapered, apical half with margin parallel through most of length, apex with a small spine slightly curved; basal half with several long macrosetae irregularly arranged and row of moderately long, slender microsetae on dorsal margin, apical half with medial row of short, stout setae. Style (Fig. 2D), in lateral view, sigmoid with three preapical setae. Connective (Fig. 2E) triangular. Aedeagus (Figs 2F–G) with preatrium nearly as long as shaft, dorsal apodeme compressed, racket-shaped in lateral view, incompletely fused to shaft; shaft short and broad, apex bifurcate, without processes; gonopore apical.

##### Material examined.

Holotype male, ARGENTINA: Jujuy, P.N. Calilegua 1600m 23°41'1"S, 64°54'0"W, 14–16 January 2008 Dietrich et al col. Malaise trap [MLP]. Paratypes: 2 males, same data as holotype [INHS].

##### Etymology.

The specific name refers to the arrangement of the five dark markings on the forewing arranged radially in the form of a flower.

##### Note.

This species closely resembles *Balera bracata* but has a short process on the pygofer and the aedeagus is wider with the apical bifurcation shorter.

### New records

#### Habralebra

Young

Habralebra Young, 1952: 33. Type species: *Protalebra nicaraguensis* (Baker), by original designation.

#### Habralebra amoena

Young

http://species-id.net/wiki/Habralebra_amoena

Fig. 1B

Protalebra amoena Baker, Psyche, vol 8, p. 404, 1899.
Habralebra amoena Young, Univ. Kansas Sci. Bull. 35, p. 34, 1952.

##### Distribution.

Brazil. New record from Argentina, Misiones.

##### Material examined.

4 males and 1 female, ARGENTINA: Misiones, Puerto Iguazú 200m 25°37'19"S, 54°32'52"W, 7 January 2008 Dietrich col. hand collected at night [2 males and 1 female in MLP, 2 males in the INHS].

## Supplementary Material

XML Treatment for Balera

XML Treatment for Balera floripara

XML Treatment for Habralebra

XML Treatment for Habralebra amoena
